# GX15–070 (Obatoclax), a Bcl-2 family proteins inhibitor engenders apoptosis and pro-survival autophagy and increases Chemosensitivity in neuroblastoma

**DOI:** 10.1186/s12885-019-6195-y

**Published:** 2019-10-29

**Authors:** Sonia Cournoyer, Anissa Addioui, Assila Belounis, Mona Beaunoyer, Carine Nyalendo, Roxane Le Gall, Pierre Teira, Elie Haddad, Gilles Vassal, Hervé Sartelet

**Affiliations:** 1 Research Center, Sainte Justine University Hospital Center, Montreal, QC Canada; 2 Department of Pathology and Cellular Biology, Université de Montréal, Montreal, QC Canada; 3 Department of Pediatric Surgery, Sainte-Justine University Hospital Center, Montreal, QC Canada; 4 Department of Pediatric Hemato-Oncology, Sainte-Justine University Hospital Center, Montreal, QC Canada; 5 Department of Pediatric Oncology, Institut Gustave Roussy, Villejuif, France; 6 Département d’anatomie et cytologie pathologiques, Institut de Biologie et Pathologie, CHU A Michallon, 38043 Grenoble cedex 09, France

**Keywords:** Bcl-2, Neuroblastoma, Apoptosis, Autophagy, GX15–070

## Abstract

**Background:**

Neuroblastoma (NB) is a frequent pediatric tumor associated with poor prognosis. The disregulation of Bcl-2, an anti-apoptotic protein, is crucial for the tumoral development and chemoresistance. Autophagy is also implicated in tumor cell survival and chemoresistance. The aim of our study was to demonstrate therapeutic efficiency of GX 15–070, a pan-Bcl-2 family inhibitor, used alone and in combination with conventional drugs or with hydroxychloroquine (HCQ), an autophagy inhibitor.

**Methods:**

Five neuroblastoma cell lines were tested for the cytotoxic activity of GX 15–070 alone or in combination with cisplatin, doxorubicin, HCQ or Z-VAD-FMK a broad-spectrum caspase inhibitor. Apoptosis and autophagy levels were studied by western-blot and FACS. Orthotopic injections were performed on NOD/LtSz-scid/IL-2Rgamma null mice that were treated with either GX 15–070 alone or in combination with HCQ.

**Results:**

Synergistic cytotoxicity was observed for the drug combination in all of the 5 neuroblastoma cell lines tested, including MYCN amplified lines and in cancer stem cells. GX 15–070 significantly increased apoptosis and autophagy in neuroblastoma cells as evidenced by increased levels of the autophagy marker, LC3-II. Inhibition of autophagy by HCQ, further increased the cytotoxicity of this combinatorial treatment, suggesting that autophagy induced by these agent plays a cytoprotective role. In vivo, GX 15–070 combined with HCQ significantly decreased the growth of the tumor and the number of distant metastases.

**Conclusions:**

Based on the synergistic effect of HCQ and GX 15–070 observed in this study, the combination of these two drugs may be utilized as a new therapeutic approach for neuroblastoma.

## Background

Neuroblastoma (NB) is a pediatric tumor of the sympathetic nervous system, with a median age of 17 months at diagnosis it is responsible for over 15% of pediatric cancer deaths [1]. In 50% of patients, it localises in in the adrenal medulla or paraspinal ganglia [1]. The NB is caracterised by it’s clinical and biological heterogeneity [1]. Indeed, according to clinical, biological and pathological tumor datas, patients with tumors classified in the low-risk group present a good survival and can show a spontanous tumor regression while in patients with high-risk NB, the long term survival is poor despite aggressive chemotherapy and radiotherapy treatments, and stem cells transplants [2]. It is therefore essential to develop new therapeutic strategies to improve the survival of patients with high-risk NB.

The concept of cancers stem cells (CSCs) appears relevant to explain the high level of metastasis, chemoresistance and unfavorable prognosis commonly observed in NB. Many studies suggest that malignant tumors contain subpopulations of cells that share characteristics with normal stem cells, that is, they are capable of self-renewal, differentiation, and initiation of tumors [3]. These CSCs are thought to be responsible for metastasis and are more resistant to chemotherapeutic agents, thereby permitting the recurrence of tumors after chemotherapy and radiotherapy [4]. CD133 has been identified as a marker of a subset of neural stem cells in the adult central nervous system as well as of glioblastoma stem-like cells [5, 6]. In NB, CD133 expression is associated with poor prognosis, chemoresistance [7] and the cells expressing this protein present stemness property [8].

Apoptosis, also called programmed cell death, is essential for the normal regulation of cell survival by suppressing superfluous or damaged cells. A defect in the apoptosic pathway is now considered to be the foundation of most cancers [9]. Apoptosis occurs in response to the disturbance of one of the two interconnected pathways known to be the extrinsinc death receptors or the intrinsic mitochondrial pathway. The mitochondrial pathway of apoptosis is regulated by Bcl-2 family proteins which regulate cell survival and is comprised of Bcl-2, Bcl-XL, Bcl-W, Bcl-2-A1, and Mcl-1. Studies show that deregulation of the Bcl-2 as primary oncogenic event (initiator) or secondary (promoter) is crucial for the tumoral development, maintenance and chemoresistance [10]. In NB, in vitro studies have demonstrated that Bcl-2 is highly expressed, and its expression is inversely correlated to the number of apoptotic cells and the level of differentiation of the tumor [11]. Also, overexpression of Bcl-2 in NB cell lines is associated with chemoresistance [12]. The high level of expression of Bcl-2 is also correlated with unfavorable histology, *MYCN* amplification and poor prognosis [12]. The analysis of the anti-apoptotic protein Bcl-2 and Mcl-1 have demonstrated a strong expression in NB [13]. It was shown that the anti-apoptotic genes such as Bcl-2 and Bcl-xL were overexpressed in CSC while the expression of pro-apoptotic genes such as Bax were downregulated in CSC of glioblastoma [14]. Also, overexpression of Bcl-2 gene contribute to the resistance of medulloblastoma CSC to radiotherapy [15]. Therefore, Bcl-2 is an attractive target in the treatment of NB. Bcl-2 inhibitor could be used alone or in combination with others drugs to potentiate treatment efficiency [16].

GX 15–070, by activating the intrinsic pathway of apoptosis, is an inhibitor of all anti-apoptotic Bcl-2 proteins. GX 15–070 inhibits specifically Bcl-XL, Bcl-2, MCL-1, Bcl-w, A1 and Bcl-B [17]. Recent study desmonstrated that NB cell lines and primary tumors are primed for death with sequestration of Bim, a direct activator of apoptosis, by either Bcl-2 or Mcl-1, providing a survival dependency that predicts the activity of Bcl-2 antagonists [18]. Analogous to its predecessor ABT-737, ABT-263 possesses high affinity for Bcl-xL, Bcl-2, and Bcl-w, but not for Mcl-1 or A1 [19]. Also in NB, Mcl-1 seems to be the principal mediator of classical Bcl-2 antagonist resistance. GX 15–070 with its large spectrum of inhibition including Mcl-1 represents a potential interest in the treatment of NB. In other tumors, in vitro preclinical studies have shown efficacy of GX 15–070 as monotherapy or in combination with other anticancer agents like in refractory mantle cell lymphoma [20] or in antiestrogen-resistant breast cancer cells [21]. Moreover, GX 15–070 selectively eradicates quiescent human leukemia stem cells [22] and radiosensitizes glioblastoma stem-like cells [23].

Another mechanism implicated in tumor cell survival and drug resistance is autophagy whitch is activated by metabolic stress [24, 25]. This lysosomal degrative pathway, caracterised by autophagosomes formation, seems to be involved in the unsuccessful therapeutic effectiveness because of tumor masses vessel, nutrient heterogenecities, and hypoxic tumor regions that undergo autophagy. Since several studies have shown that autophagy is crucial as a survival mechanism in different tumors with defects in the apoptotic pathway, the modulation of these pathways could be an interesting avenue for improvement of NB treatments [26]. Autophagy contributes to modulating the cytotoxicities of Bcl-2 homology domain-3 mimetics [27]. Beclin 1, a Bcl-2 homology 3 (BH3) domain only protein is an essential initiator of autophagy. In addition, Beclin 1 is a key determining factor as to whether cells undergo autophagy or apoptosis [28]. Beclin 1 has been shown to interact via its BH3 domain with Bcl-2 family members. The dual role of Bcl-2 and Bcl-xL in inhibiting both apoptosis and autophagic-associated cell death makes these proteins ideal chemotherapeutic targets. BH3 mimetics, GX 15–070 and ABT-737, disrupt the Bcl-2-Beclin1 interaction. GX15–070 induces pro-survival autophagy in head and neck squamous cell carcinoma cells [29] whereas the combination of GX15–070 with chloroquine, a specific autophagy inhibitor, results in synergistic cytotoxicity against pancreatic cancer cells [20].

The aim of our study was to evaluate the activity of Obatoclax against NB cells used in monotherapy or in combination with conventional drugs or with an autophagy inhibitor, hydroxychloroquine (HCQ).

## Methods

### Cell lines

Five distinct established human NB cell lines were used for this study. SK-N-DZ (ATCC® CRL-2149) and SK-N-FI (ATCC® CRL-2142) were purchased from American Type Culture Collection (VA, USA), SJNB-10 from St-Jude Research Hospital in Memphis (SJNB-10, RRID:CVCL_1441, obtained from T. Look) and IGR-N91 and IGR-NB8 kind gifts from Dr. Benard (Institut Gustave Roussy Paris). SJNB-10, SK-N-DZ, IGR-N91 and IGR-NB8 present a *MYCN* amplification. All cell lines were grown in Dulbecco’s modified eagle’s medium (DMEM) supplemented with 1% penicillin/streptomycin (Multicell), 1.25 μg/mL fungizone (Gibco) and 10% fetal bovine serum and maintained at 37 °C in a humidified atmosphere composed of 5% CO_2_. The cell lines were tested for Mycoplasma infection using PCR. When the cells reached 60–70% of confluence, cells were treated in vivo with different concentrations of drugs as a single agent or combined for 24–48 h at 37 °C: vehicle (polyethylene glycol 300 (96.2%) and polysorbate 20 (3.8%)) or Obatoclax (GX 50–170) or Cisplatin (Cis) or doxorubicin (Dox). GX 50–170 were kindly provided by Gemin X (Montreal, Canada).

### CSCs sorting by flow cytometry

We sorted CSCs by flow cytometry. SK-N-DZ cells were stained with APC (allophycocyanin)-conjugated anti-human CD133/2 antibody (Miltenyi Biotec, Auburn, CA) according to the manufacturer’s instructions. Briefly, cells were stained with the antibody APC-labeled in the dark at 4 °C for 30 min in a 2% FBS-PBS 1X stain buffer and filtered in a 35 μm nylon mesh incorporated into the tube cap (Beckman Dickinson Falcon™, Canaan, CT). After, CD133 sorted cells (CD133^high^ CSCs and CD133^low^) were each isolated in an 80% FBS-PBS 1X buffer using the FACS Aria (BD Biosciences, Mississauga, ON). Gating was established using an APC-conjugated anti-human IgG2b (Miltenyi Biotec, Auburn, CA). The specificity of the selection was evaluated by flow cytometry with the FACS Aria with CD133/2 antibody and vimentin-PE (Miltenyi Biotec, Auburn, CA). Selected cell lines showed CD133 expression in CD133^high^ population ranging from 85 to 95% and vimentin expression. On the other hand, CD133l^ow^ population, revealed CD133 expression ranging from 1.29% to 14,6% and poor vimentin expression.

### Cell viability assays

Cell viability was determined by the MTT (3-[4, 5-dimethylthiazol-2-yl]-2,5-diphenyl tetrazolium bromide) reduction assay (Promega, Madison, WI) according to the manufacturer’s instructions. Cells including SK-N-DZ cells sorted for CD133 (CD133^high^ and CD133^low^) were plated in 96-well culture plate at a density of 5 × 10^3^/well overnight. To test the effectiveness of GX 70–150 inhibition of cell growth, cells were exposed to increased concentration of GX 50–170 (0.05–5 μM) for 24-48 h. The compound concentration resulting in 50% inhibition of cell viability (IC_50_) was determined using GraphPad software. To determine whether Cis or Dox would increase cell sensitivity to GX 50–170, cells were treated with varying concentration of Cis (0.15–75 μg/mL) and Dox (0.005–5 μg/mL) with or without GX 50–170 for 48 h. The mean percentage of cell survival relative to that of vehicle-treated cells was estimated from data of three individual experiments performed by triplicate.

To test the importance of caspase-mediated apoptosis in cell death induced by GX 50–170, SK-N-DZ and IGR-NB8 cells were treated with increased concentration of GX 50–170 (0.05–5 μM) for 48 h, with or without Z-VAD-FMK (carbobenzoxy-valyl-alanyl-aspartyl-[O-methyl]- fluoromethylketone) 20 μM, a cell-permanent pan-caspase inhibitor, that irreversibly binds to the catalytic site of caspase proteases. In the same way, to evaluate the effect of autophagy activation induced by GX 50–170, cell lines were treated with increased concentrations of GX 15–070 or combined with (30 μM) of hydroxychloroquine (HCQ), a specific inhibitor of the late phase of autophagy, prepared in PBS for 48 h.

### Protein extraction and Western blotting

Cells previously treated were lysed with lysis buffer (10 mM Tris-HCl, pH 7.4, 150 mM NaCl, 1 mM EDTA, 1 mM EGTA, 1% Triton X-100, 0.5% Nonidet P-40, 1 mM sodium orthovanadate and 1 mM sodium fluoride), 30 min at 4 °C. Protein concentration was measured using the BCA assay kit (Pierce, Rockford, IL). Equal amounts of cell lysate protein were resolved by SDS-PAGE. After electrophoresis, proteins were transferred to PVDF membrane (Millipore, Bedford, MA). Membranes were blocked for 1 h (TBS buffer (20 mM Tris-HCl, pH 7.4, 150 mM NaCl) containing 3% bovine serum albumin)) and then incubated with primary antibodies. The primary antibodies used for this study were against Bcl-2 (ab692, abcam), LC3B (ab51520, abcam), Beclin 1 (ab55878, abcam), Mcl-1 (sc20679, Santa Cruz biotechnology), Bcl-XL (2764, cell signaling), Bax (32,503, abcam) Cleaved Caspase-3 (9664, Cell Signaling Technology), ATG5 (2630, Cell Signaling Technology), CD133 (1/2000, Abcam, Cambridge, MA and β-actin (sc-69,879, Santa Cruz Biotechnology). Membranes were washed and incubated with horseradish peroxidase-conjugated secondary anti-rabbit or anti-mouse antibodies. Antibody binding was visualized by chemiluminescence (ECL, Perkin Elmer, Waltham, MA) before exposure of the membrane to a photosensitive film. Analysis by densitometry using Kodak ID 3.6 software allowed us to calculate the relative expression of caspase 3 and LC3 in treated cells.

### Detection of cell death by flow cytometry

Detection of cell death was performed using an Annexin V/7-AAD (7-aminoactinomycin D) staining with the ApoAlert Annexin V-FITC Apoptosis Kit (Clontech Laboratories Inc., Mountain View, CA) according to the manufacturer’s instructions. Briefly, 1 × 10^6^ cells were treated with increased GX 15–070 concentrations (0.05–5 μM) for 48 h. Then, cells were dissociated with 0.5 mM EDTA and stained with Annexin V-FITC and 7-AAD in the dark for 30 min at 4 °C. Cells were analyzed by LRSFortessa (BD Biosciences, Mississauga, ON).

### Animals

Female and male NOD/LtSz-scid/IL-2Rgamma null (NSG) mice (6–8 weeks old) were obtained from the Jackson Laboratory (Bar Harbor, ME), bred in the animal facility under pathogen-free conditions and fed with autoclaved water and diet. Experiments were carried out under the conditions established by the Good Laboratory Practices for Animal Research institutional committee (Permit Number: 467) of the Sainte-Justine University Hospital Center. All mice were housed in ventilated cages under standard conditions of controlled temperature and humidity, and exposed to a 12-hourly light/dark cycle. NSG were established in 6- to 8-week-old mice by injection into the left adrenal gland of five NSG mice, respectively, under general anesthesia with 2% isofluran/O_2_. A clinical analysis (weight, vital signs, and behavior) was performed once a week. Mice injected orthotopically were treated intraperitonealy (*i.p*) with GX 15–070 alone (*n* = 5) or in combination with HCQ (n = 5), delivered at 3 mg/kg and 60 mg/kg or with PBS used as negative controls (n = 5) respectively for 5 days (from day 18 to 22 post NB cells injection). After 4 weeks, mice were sacrificed in a CO_2_ chamber. A necropsy was done and the left adrenal gland (injection site) as well as liver, lungs, brain and femur (all potential sites of metastasis) were removed, fixed in 10% phosphate-buffered formalin and embedded in paraffin. Tumor size was determined and volumes were measured by caliper and calculated using the formula: Tumor volume = (length x width^2^)/2.

### Statistical analysis

All data are represented as the mean ± SEM. Cell survival statistical analyses were performed using Student’s t-test with Graphpad Prism software (Version 5.00). Tumor volume statistics were analyzed by one-way analysis of variance (ANOVA) followed by bonferroni post-test. *P* < 0.05 was considered statistically significant.

Synergism was also evaluated by median dose effect analysis using the Chou–Talaly method and a software program (CalcuSyn, Biosoft, Ferguson, MO) [30]. Cells were treated with Cis or Dox or HCQ and obatoclax at fixed concentration ratios for 48 h and cell death was monitored by MTT. Combination index (CI) values and isobolograms were computed using CalcuSyn software. CI values less than 1.0 denote synergism.

## Results

### GX 15–070 used in monotherapy in NB cells

**Fig. 1:**
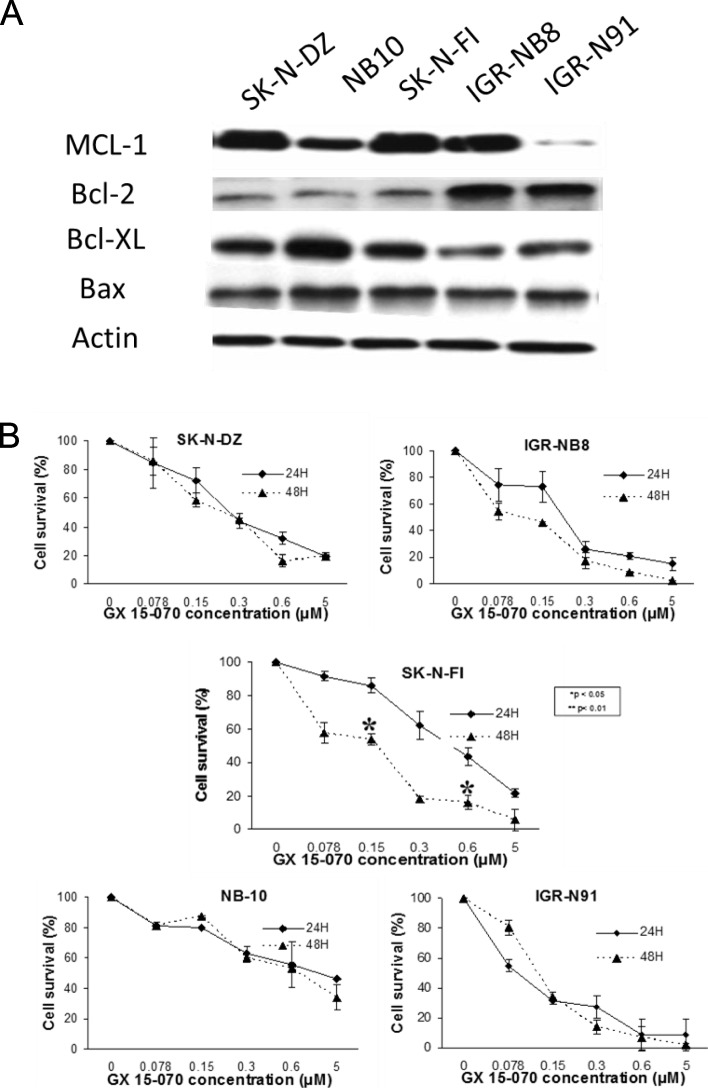
**a** Basal level expression of important pro and antiapoptotic BCL-2 family proteins in human neuroblastoma. β-actin served as loading control. **b**. The effect of GX 15–070 used in monotherapy in NB cell viability in vitro. IGR-NB8, IGR-91, NB-10, SK-N-DZ and SK-N-FI cells treated 24 and 48 h with increasing concentrations of GX 15–070 (0.078 μM, 0.15 μM, 0.3 μM, 0.6 μM, and 5 μM).were added to the medium for 24 and 48 h and cell viability determined by the standard methyl-thiazoldiphenyl tetrazolium (MTT) assay. Each value represents the mean ± SEM (*n* = 3)

The basal levels of important pro and antiapoptotic BCL-2 family proteins were assessed by western blotting (Fig. 1a). IGR-N91 expressed low levels of MCL-1 protein as compared to the aothers cell lines. Notably, all the cell lines expressed relatively higher levels of at least two of the three predominant antiapoptotic BCL-2 family proteins. All NB cell lines exposed for 24 or 48 h to GX 15–070 exhibited dose-response curves characteristic (Fig. 1b) including those with MYCN amplification (NB-10, SK-N-DZ, IGR-N91 and IGR-NB8). They showed IC50 inferior to 1 μM and were considered sensitive to this drug used in monotherapy (Table 1). For two cell lines (IGR-NB8 and IGRN91), IC50 was less than 100 nM. The survival of SK-N-FI cell line (without MYCN amplification) is significantly decreased after a longer exposure (48 h) (Fig. 1b) while for the other cell lines, the sensitivity of the cells was time independent.

**Table 1:** IC_50_ of NB cell lines treated with GX 15–070

NB cell lines	IC_50_ (μM)	IC_50_ (μM)
	24 h	48 h
SK-N-DZ	0,213	0,164
SK-N-FI	0,379	0,164
NB-10	0,177	0,309
IGR-NB8	0,188	0,090
IGR-N91	0,064	0,114

### GX 15–070 induced neuroblastoma apoptosis

**Fig. 2:**
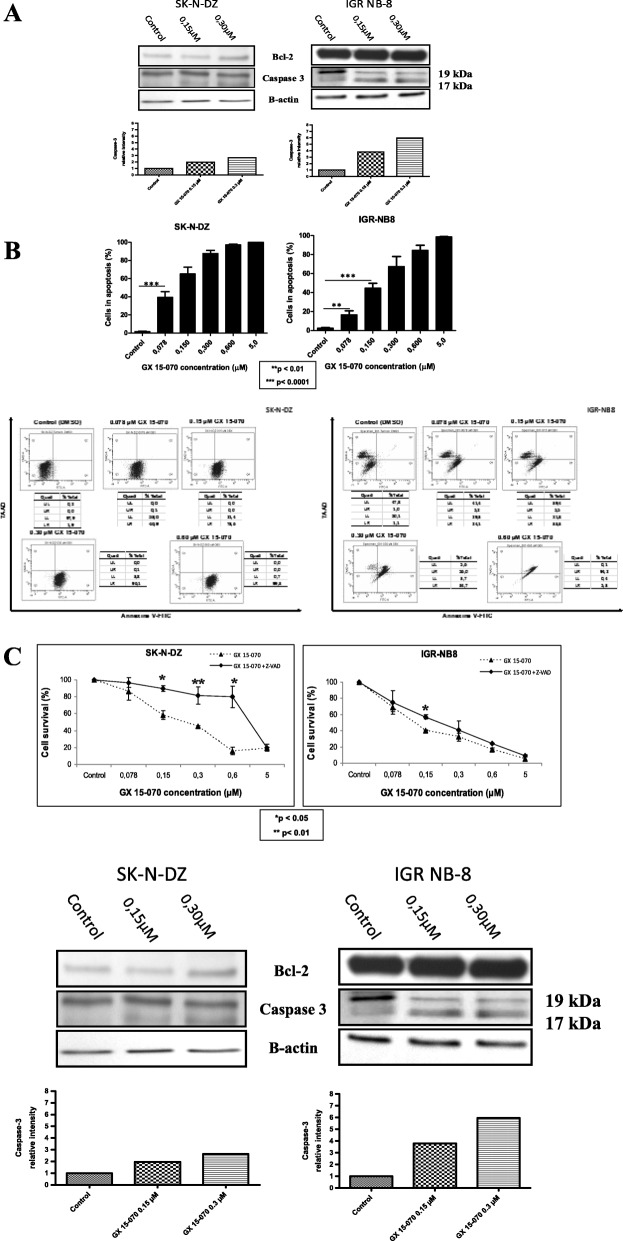
GX 15–070 induced cell death via apoptosis pathway. **a** Western blotting demonstrated Bcl-2 and cleaved caspase-3 expressions in SK-N-DZ and IGR-NB8 cells treated with GX 15–070. **b** SK-N-DZ and IGR-NB8 cells treated with different concentrations of GX 15–070 ranging from 0.078 to 5 μM for 48 h. Induction of apoptosis detected by flow cytometric analysis using Annexin V-FITC and 7-AAD staining. **c** 5X10^3^ SK-N-DZ and IGR-NB8 cells treated with different concentrations of GX 15–070 ranging from 0.05 μM to 5 μM in combination with Z-VAD-FMZ 20 μM for 48 h. Cell viability determined by MTT assays. Viability was calculated as the percentage of living cells in treated cultures compared to those in control cultures. Each value represents the mean ± SEM (n = 3)

To verify if GX 15–070 induces cell death by caspase dependant apoptosis, western blots were performed on two cell lines (SK-N-DZ and IGR NB-8) treated with GX 15–070. In both cell lines, there was a correlation between the level of cleaved caspase 3 and the concentration of GX 15–070 used in monotherapy (Fig. 2a). The analysis by Annexin V/7-AAD staining confirmed that GX 15–070 induces increased apoptosis in a dose dependent manner (early and late apoptosis) (*p* < 0.0001) (Fig. 2b). The difference with control was already significant in both cell lines at a very low concentration of GX 15–070 (75 nM). Subsequent treatment of both cell lines with the pan-caspase inhibitor, Z-VAD-FMK, significantly inhibited the cell death indicating that apoptosis is mainly caspase-mediated (Fig. 2c). This activated apoptosis was not correlated with modification of Bcl-2 expression (Fig. 2a).

### Autophagy is also activated by GX 15–070 treatment

**Fig. 3:**
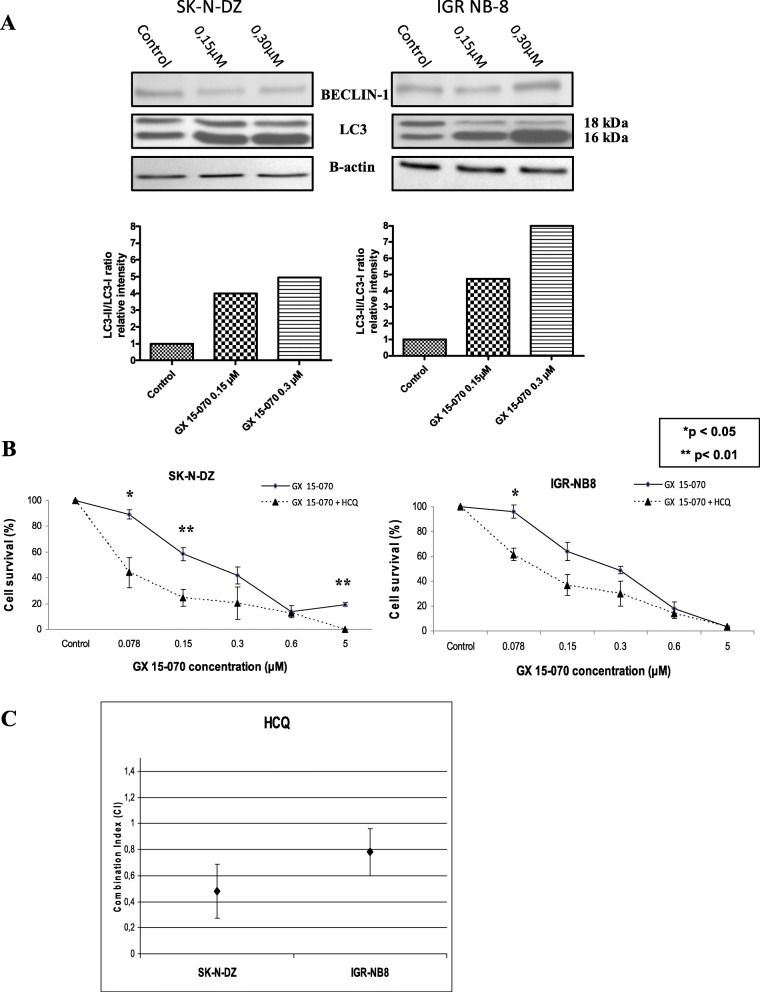
Autophagy pathway modulated by GX 15–070 treatment. **a** Immunoblotting analyses were performed on protein lysates from SK-N-DZ and IGR-NB8 cells treated with GX 15–070. Anti-LC3 and Beclin 1 antibodies were used. β-actin was used as a loading control.. **b** Cell viability of 5X10^3^ SK-N-DZ and IGR-NB8 cells treated with different concentrations of GX 15–070 ranging from 0.078 to 5 μM in combination with HCQ at (30 μM) for 48 h was determined by MTT assays. Viability was calculated as the percentage of living cells in treated cultures compared to those in control cultures. Each value represents the mean ± SEM (n = 3). **c** Synergistic effect of GX 15–070 and HCQ on cell growth in vitro. Cells were treated with increasing concentrations of drugs either alone or concurrently at their equipotent molar ratio and combination indices (CIs) calculated by the method of Chou and Talaly (CI calculates synergism,0.8; additivity between .0.8 and,1.2; antagonism .1.2. All values are given as mean + SD of at least 3 independent experiments)

The level of autophagy in cells treated with GX 15–070 was then evaluated by the study of LC3 protein cleavage by western blots (Fig. 3a). The LC3-II/LC3-I ratio increased in SK-N-DZ and IGR-NB8 cells treated with GX 15–070 compared to control (Fig. 3a). The specific inhibition of the late autophagy by HCQ significantly increased cells sensitivity to GX 15–070 (Fig. 3b).

### The cancer stem cells were less sensitive to GX 15–070

**Fig. 4:**
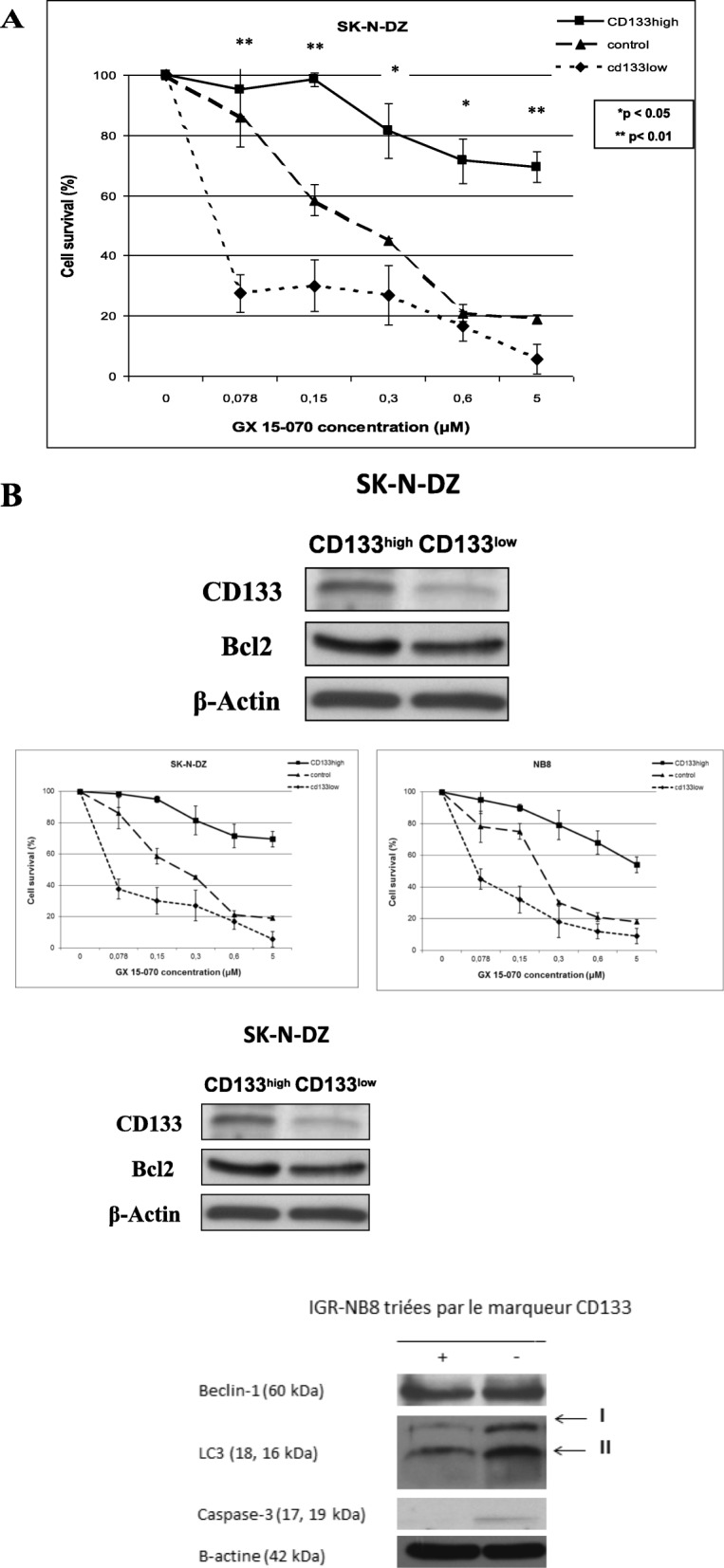
The effect of GX 15–070 used alone in NB cancer stem cells viability (**a**) and expression of Bcl-2 in NB CSCs (**b**). Cells viability of CD133 sorted SK-N-DZ cells treated for 48 h with GX 15–070 were measured using the standard MTT (The statistical difference between CD133^*high*^ and CD133^*low*^ was calculated with Fisher’s exact test) (**a**)**.** Immunoblotting analyses were performed on protein lysates from SK-N-DZ cells. Anti-CD133 and Bcl-2 antibodies were used (**b**)

SK-N-DZ cells were sorted into CD133^high^ and CD133^low^ populations and expressed two CSC markers of CSCs: CD133 and vimentin [31]. They were exposed for 48 h to varying concentration of GX 15–070. CD133^high^ cells were found to be more resistant to treatment than their matched CD133^low^ counterparts (Fig. 4a). This data was in correlation with the higher expression of Bcl2 in CSCs (Fig. 4b).

### GX 15–070 used in combined therapy in NB cells

**Fig. 5:**
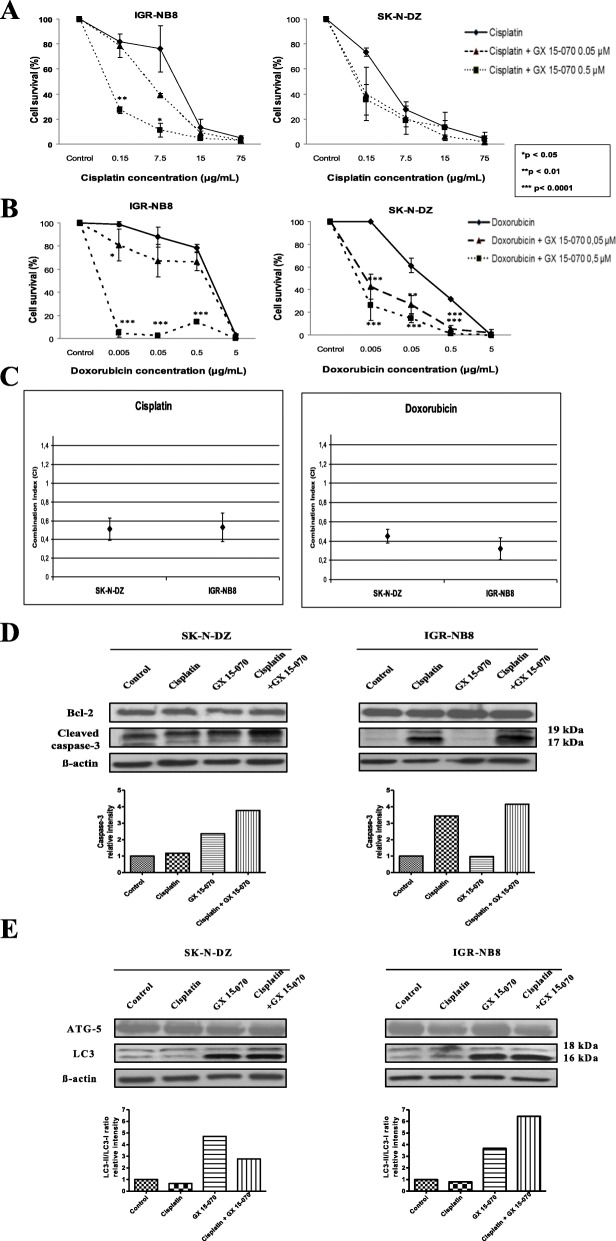
The combination effect of GX 15–070 and cisplatin or doxorubicin in NB cells. 5X10^3^ SK-N-DZ and IGR-NB8 cells treated with GX 15–070 IC_50_ calculated at 48 h post-treatment and different concentrations of cisplatin (**a**) and doxorubicin (**b**) ranging from 0.015 to 75.0 μg/mL and from 0.005 to 5.0 μg/mL respectively during 48 h. Viability was calculated as the percentage of living cells in treated cultures compared to those in control cultures. Each value represents the mean ± SEM (n = 3). Western blotting showed cleaved caspase-3 (**c**) and LC3I and LC3II expression (**d**) in SK-N-DZ and IGR-NB8 cells treated with 0.05 μM GX 15–070 or in combination with cisplatin. (**e**) Synergistic effect of GX 15–070 and cisplatin or doxorubicin on cell growth in vitro. Cells were treated with increasing concentrations of drugs either alone or concurrently at their equipotent molar ratio and combination indices (CIs) calculated by the method of Chou and Talaly

Co-administration of GX 15–070 concentrations as low as 0.05 μM significantly increased cell death, when it is combined with Cis and Dox, chemotherapeutic agents classically used in NB treatments in both cell lines used in the present study (Fig. 5a and b). Interestingly, the presence of 0.05 μM of GX 15–070 significantly decrease the IC50 for Dox and Cis in both cell lines (Table 2 and Table 3). The decrease in cell survival associated with the Cis and GX 15–070 combination is associated with caspase mediated apoptosis clearly demonstrated by the study of caspase 3 cleavage but without modifying the level of BCL-2 expression (Fig. 5d). More interestingly, this association clearly increased the level of autophagy detected by LC3II/LC3I ratio but did not modify ATG5 expression (Fig. 5e).

**Table 2:** IC_50_ of NB cell lines treated with GX 15–070 and cisplatin

NB cell lines	IC_50_ (μM)	IC_50_ (μM)	IC_50_ (μM)
	Cis^a^	Cis^a^ + 0,05 μM GX 15–070	Cis^a^ + 0,5 μM GX 15–070
SK-N-DZ	0,409	0,078	0,055
IGR-NB8	13,01	4439	0,043

^a^*Cis* cisplatin

**Table 3:** IC_50_ of NB cell lines treated with GX 15–070 and doxorubicin

NB cell lines	IC_50_ (μM)	IC_50_ (μM)	IC_50_ (μM)
	Dox^a^	Dox^a^ + 0,05 μM GX 15–070	Dox^a^ + 0,5 μM GX 15–070
SK-N-DZ	0,110	0,002	3283^a^10^− 4^
IGR-NB8	1183	0,982	8565^a^10 ^− 11^

^a^*Dox* doxorubicin

Median Dose Effect analysis was used to evaluate synergism. Median Dose Effect analysis revealed Combination Index values < 1.0, indicating synergistic interactions. Synergistic effects of the combination of GX 15–070 with Cis or Dox or HCQ on cell death were observed in both cell lines. The combination indices (CI) of GX 15–070 with Cis were synergistic in SK-N-DZ and IGR-NB8 (CI 0.51 and CI 0.53, respectively) (Fig. 5c), those with Dox were synergistic in SK-N-DZ and IGR-NB8 (CI 0.45 and CI 0.32, respectively) (Fig. 5c), and those with HCQ were synergistic in SK-N-DZ and IGR-NB8 (CI 0.48 and CI 0.78, respectively) (Fig. 3c).

### Effect of GX 15–070 used alone or in combination with HCQ on tumor volume

**Fig. 6:**
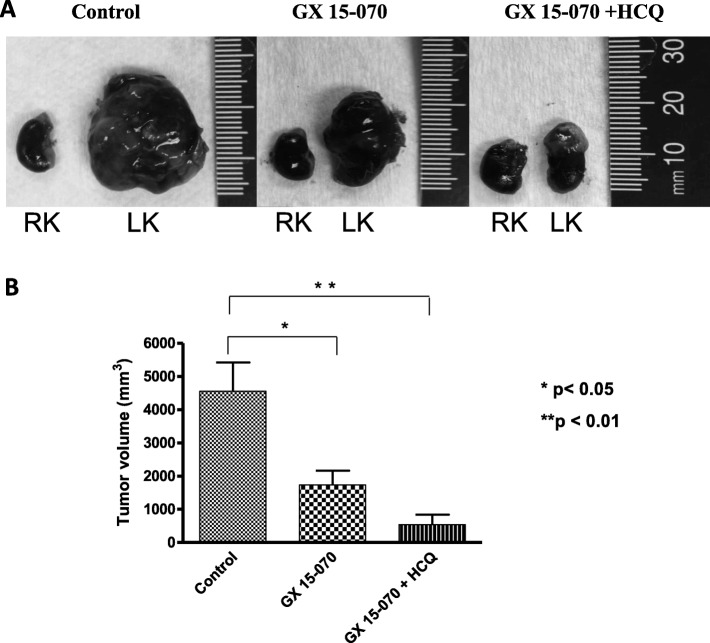
Primary tumor sizes and metastases decreased with GX 15–070 and HCQ treatments. **a** Adrenal gland of an NSG mouse was injected with 1*10^6^ SK-N-DZ cells. On day 18 after orthotopic injection, a dose of GX 15–070 (3 mg/kg/day) was injected *i.p. into mice* alone or in combination with HCQ (60 mg/kg/day) for 5 days. Control mice were injected with saline. Right adrenal gland wasn’t injected. **b** At necropsy, tumor volumes were calculated. LK: Left kidney; RK: Right kidney

The in vivo study confirmed the sensitivity of the tumor cells to GX 15–070. Four weeks after orthotopic injections of SK-N-DZ cells in mice adrenal gland, the size of the tumor was significantly less in mice treated with GX 15–070 or with GX 15–070 combined with HCQ than control (*p* < 0.05 and *p* < 0.01 respectively) (Fig. 6a, b and Table 4). Moreover, the liver and lung metastases were less frequent in mice treated with GX 15–070 than in control and were absent in mice treated with the synergistic drugs (GX 15–070 and HCQ) (Table 4).

**Table 4:** Orthotopic injections in NOD/SCID/IL2Rcc-null mice

Cell line	Number of cells engrafted	Treatments	Primary Tumor Volume (mm^3^)	Metastases	
				Liver	Lung
SK-N-DZ	1 000 000	Control	3 559	5/5	5/5
		GX 15-070	1 630	1/5	5/5
		GX 15-070 + HCQ^a^	540	0/5	0/5

^a^HCQ: Hydroxychloroquine

## Discussion

Increased expression of the anti-apoptotic members of the BCL-2 family proteins correlates with chemotherapy resistance in NB [13]. These major anti-apoptotic proteins are known to inhibit cell apoptosis, and therefore targeting them may restore the capacity of NB cells to undergo apoptosis. The present data demonstrate that GX 15–070, has an anti-tumor effect in NB which was shown when cell lines were treated with GX 15–070 monotherapy. GX 15–070 treatment is time dependant with significantly decreased IC_50_ after 72 h (ranging from 0.014 to 1.449 μM). This variability observed in our data has similarities with studies done in multiple myeloma cells where the IC_50_ of 16 cell lines ranged from 0.052 to 1.100 μM after a 72 h GX 15–070 treatment [20]. This group showed that cell sensitivities to GX 15–070 treatment correlated with protein levels of different Bcl-2 protein members. In fact, cells that downexpressed Bcl-xL were the most sensitive, while those strongly expressed Mcl-1, Bcl-2 and Bcl-xL were more resistant to GX 15–070 treatment. A direct relationship was also demonstrated between the dose-response curves and the absence or near absence of Bak protein in the cell. Therefore, GX 15–070 used alone will be of greater efficiency in tumors that have reduced, absent or inactivated expression of Bcl-xL and Bak proteins [20]. In NB, a similar study was carried out with another inhibitor of Bcl-2, ABT-737, a mimetic molecule of proapoptotic BH3-only, which binds specifically Bcl-2, Bcl-xL and Bcl-w but has no affinity for Bcl-2-A1 and Mcl-1 [25]. Their results showed cellular sensitivity varied from 0.1 μM to 100 μM. This reduced sensitivity in NB cell lines was also found in a second study involving the ABT-737 monotherapy [32]. Due to its cellular heterogeneity, NB treatment should be more effective with GX 15–070 rather than ABT-737 because of the potential to inhibit all the antiapoptotic proteins, including Mcl-1 [33]. The dose-dependent cytotoxic effects of GX 15–070 and the heterogeneities underlying each tumor suggests that the most convincing therapeutic effects would be a combination of GX 15–070 with other anti-cancer agents [34, 35].

We also observed a significant decrease cell survival when NB cell lines were treated with GX 15–070 in combination with Cis. This platinum-based agent that forms bridges between the DNA strands activates DNA repair mechanisms and, if the repair is too large, apoptosis is activated through the mitochondrial pathway [36]. Crawford and collegues showed a significant decrease in resistance when small lung cancer cells, already resistant to cisplatin, were treated with a combination of Cis and 0.5 μM of GX 15–070 [37]. In NB, a combination of ABT-737 with different chemotherapeutic agents like Cis, vincristine, etoposide or Dxr was done. All these agents activated the intrinsic apoptotic pathway and their combination with Bcl-2 inhibitor synergistically increased cell line toxicity [36, 38]. Therfore, it seems that Bcl-2 inhibitors are able to sensitize tumor cells to conventional chemotherapeutic agents and reverse the resistance caused by the disregulation of the apoptotic machinery.

Our study also showed that GX 15–070 induced apoptosis in NB cell lines. Moreover, when this pathway is blocked, cell survival is restored and significantly increased even if cells are treated with GX 15–070. A previous study done in acute myeloid leukemia cells showed that GX 15–070 induced apoptosis by many mitochondrial pathways [39]. On isolated mitochondria, GX 15–070 treatments significantly increased the release of cytochrome C in the cytoplasm [39]. Intersingly, this study demonstrated that the absence of Bax and Bak doesn’t eliminate completely the apoptosis induced by GX 15–070 unlike its counterpart ABT-737 [40]. These results suggest that GX 15–070 has multiple targets and induce cell death by other additional mechanisms.

Multiple studies have demonstrated the role of GX 15–070 in autophagy [39, 41–44]. There is a spatial organization of autophagy and apoptosis control in which BH3-only proteins exert two independent functions. On the one hand, they can induce apoptosis, by (directly or indirectly) activating the mitochondrion-permeabilizing function of pro-apoptotic multidomain proteins from the Bcl-2 family. On the other hand, they can activate autophagy by liberating Beclin 1 from its inhibition by Bcl-2/Bcl-X(L) at the level of the endoplasmic reticulum [45]. Our study demonstrated that autophagy is increased when NB cells are treated with GX 15–070 alone or in combination with Cis. Also, we observed a significant decrease in the primary tumor volume when mice are treated with GX 15–070 alone or in combination with an autophagy inhibitor, HCQ, compared to controls. A study in prostate cancer showed a significant decrease in the tumor growth when mice are treated with dual inhibition of apoptosis and autophagy [26].

## Conclusions

Our study supports the interest of using GX 15–070 alone or in combination with other conventional chemotherapeutic agents in the treatment of NB. Our data also illustrates that activating apoptosis and inhibiting autophagy pathways simultaneously have a synergistic antiproliferative potential and have to be considered in further studies surrounding NB treatment.

## Data Availability

The datasets used and/or analyzed during the current study are available from the corresponding author on request.

## Abbreviations

APC: Allophycocyanin
CI: Combination index
Cis: Cisplatin
CSC: Cancers stem cell
DMEM: Dulbecco’s modified eagle’s medium
Dox: Doxorubicin
HCQ: Ydroxychloroquine
NB: Neuroblastoma
PVDF: Polyvinylidene difluoride

## References

[1] Maris JM (2010). Recent advances in neuroblastoma. N Engl J Med.

[2] Zage PE, Louis CU, Cohn SL (2012). New aspects of neuroblastoma treatment: ASPHO 2011 symposium review. Pediatr Blood Cancer.

[3] Visvader JE, Lindeman GJ (2008). Cancer stem cells in solid tumours: accumulating evidence and unresolved questions. Nat Rev Cancer.

[4] Reya T, Morrison SJ, Clarke MF, Weissman IL (2001). Stem cells, cancer, and cancer stem cells. Nature.

[5] Singh SK, Hawkins C, Clarke ID, Squire JA, Bayani J, Hide T, Henkelman RM, Cusimano MD, Dirks PB (2004). Identification of human brain tumour initiating cells. Nature.

[6] Singh SK, Clarke ID, Terasaki M, Bonn VE, Hawkins C, Squire J, Dirks PB (2003). Identification of a cancer stem cell in human brain tumors. Cancer Res.

[7] Sartelet H, Imbriglio T, Nyalendo C, Haddad E, Annabi B, Duval M, Fetni R, Victor K, Alexendrov L, Sinnett D, Fabre M (2012). Vassal G.CD133 expression is associated with poor outcome in neuroblastoma via chemoresistance mediated by the AKT pathway. Histopathology.

[8] Cournoyer S, Nyalendo C, Addioui A, Belounis A, Beaunoyer M, Aumont A, Teira P, Duval M, Fernandes K, Fetni R (2012). Genotype analysis of tumor-initiating cells expressing CD133 in neuroblastoma. Genes Chromosomes Cancer.

[9] Hanahan D, Weinberg RA (2000). The hallmarks of cancer. Cell.

[10] Adams JM, Cory S (2007). The Bcl-2 apoptotic switch in cancer development and therapy. Oncogene.

[11] Castle VP, Heidelberger KP, Bromberg J, Ou X, Dole M, Nuñez G (1993). Expression of the apoptosis-suppressing protein bcl-2, in neuroblastoma is associated with unfavorable histology and N-myc amplification. Am J Pathol.

[12] Dole M, Nuñez G, Merchant AK, Maybaum J, Rode CK, Bloch CA, Castle VP (1994). Bcl-2 inhibits chemotherapy-induced apoptosis in neuroblastoma. Cancer Res.

[13] Goldsmith KC, Lestini BJ, Gross M, Ip L, Bhumbla A, Zhang X, Zhao H, Liu X, Hogarty MD (2010). BH3 response profiles from neuroblastoma mitochondria predict activity of small molecule Bcl-2 family antagonists. Cell Death Differ.

[14] Liu G, Yuan X, Zeng Z, Tunici P, Ng H, Abdulkadir IR, Lu L, Irvin D, Black KL, Yu JS (2006). Analysis of gene expression and chemoresistance of CD133+ cancer stem cells in glioblastoma. Mol Cancer.

[15] Yu CC, Chiou GY, Lee YY, Chang YL, Huang PI, Cheng YW, Tai LK, Ku HH, Chiou SH, Wong TT (2010). Medulloblastoma-derived tumor stem-like cells acquired resistance to TRAIL-induced apoptosis and radiosensitivity. Childs Nerv Syst.

[16] Besançon OG, Tytgat GA, Meinsma R, Leen R, Hoebink J, Kalayda GV, Jaehde U, Caron HN, van Kuilenburg AB (2012). Synergistic interaction between cisplatin and gemcitabine in neuroblastoma cell lines and multicellular tumor spheroids. Cancer Lett.

[17] Zhai D, Jin C, Satterthwait AC, Reed JC (2006). Comparison of chemical inhibitors of antiapoptotic Bcl-2-family proteins. Cell Death Differ.

[18] Goldsmith KC, Gross M, Peirce S, Luyindula D, Liu X, Vu A, Sliozberg M, Guo R, Zhao H, Reynolds CP (2012). Mitochondrial Bcl-2 family dynamics define therapy response and resistance in neuroblastoma. Cancer Res.

[19] Tse C, Shoemaker AR, Adickes J, Anderson MG, Chen J, Jin S, Johnson EF, Marsh KC, Mitten MJ, Nimmer P (2008). ABT-263: a potent and orally bioavailable Bcl-2 family inhibitor. Cancer Res.

[20] Goy A, Hernandez-Ilzaliturri FJ, Kahl B, Ford P, Protomastro E, Berger M (2014). A phase I/II study of the pan Bcl-2 inhibitor obatoclax mesylate plus bortezomib for relapsed or refractory mantle cell lymphoma. Leuk Lymphoma..

[21] Schwartz-Roberts JL, Shajahan AN, Cook KL, Wärri A, Abu-Asab M, Clarke R (2013). GX15-070 (obatoclax) induces apoptosis and inhibits cathepsin D- and L-mediated autophagosomal lysis in antiestrogen-resistant breast cancer cells. Mol Cancer Ther.

[22] Lagadinou ED, Sach A, Callahan K, Rossi RM, Neering SJ, Minhajuddin M, Ashton JM, Pei S, Grose V, O'Dwyer KM, Liesveld JL (2013). BCL-2 inhibition targets oxidative phosphorylation and selectively eradicates quiescent human leukemia stem cells. Cell Stem Cell.

[23] Berghauser Pont LM, Spoor JK, Venkatesan S, Swagemakers S, Kloezeman JJ, Dirven CM, van der Spek PJ, Lamfers ML, Leenstra S (2014). The Bcl-2 inhibitor Obatoclax overcomes resistance to histone deacetylase inhibitors SAHA and LBH589 as radiosensitizers in patient-derived glioblastoma stem-like cells. Genes Cancer.

[24] Levine B, Kroemer G (2008). Autophagy in the pathogenesis of disease. Cell.

[25] White E, DiPaola RS (2009). The double-edged sword of autophagy modulation in cancer. Clin Cancer Res.

[26] Saleem A, Dvorzhinski D, Santanam U, Mathew R, Bray K, Stein M, White E, DiPaola RS (2012). Effect of dual inhibition of apoptosis and autophagy in prostate cancer. Prostate.

[27] Yu L, Liu S (2013). Autophagy contributes to modulating the cytotoxicities of Bcl-2 homology domain-3 mimetics. Semin Cancer Biol.

[28] Marquez RT, Xu L (2012). Bcl-2: Beclin 1 complex: multiple, mechanisms regulating autophagy/apoptosis toggle switch. Am J Cancer Res.

[29] Yazbeck VY, Li C, Grandis JR, Zang Y, Johnson DE (2014). Single-agent obatoclax (GX15-070) potently induces apoptosis and pro-survival autophagy in head and neck squamous cell carcinoma cells. Oral Oncol.

[30] Chou TC, Talaly P (1977). A simple generalized equation for the analysis of multiple inhibitions of Michaelis-Menten kinetic systems. J Biol Chem.

[31] Heng X, Naiditch J, Czurylo M, Jie C, Lautz T, Clark S, Jafari N, Qiu Y, Chu F, Madonna MB (2013). Differential effect of long-term drug selection with doxorubicin and vorinostat on neuroblastoma cells with cancer stem cell characteristics. Cell Death Dis.

[32] Lock R, Carol H, Houghton PJ, Morton CL, Kolb EA, Gorlick R, Reynolds CP, Maris JM, Keir ST, Wu J (2008). Initial testing (stage 1) of the BH3 mimetic ABT-263 by the pediatric preclinical testing program. Pediatr Blood Cancer.

[33] Konopleva M, Contractor R, Tsao T, Samudio I, Ruvolo PP, Kitada S, Deng X, Zhai D, Shi YX, Sneed T (2006). Mechanisms of apoptosis sensitivity and resistance to the BH3 mimetic ABT-737 in acute myeloid leukemia. Cancer Cell.

[34] Vogler M, Dinsdale D, Dyer MJ, Cohen GM (2009). Bcl-2 inhibitors: small molecules with a big impact on cancer therapy. Cell Death Differ.

[35] Beauchamp EM, Platanias LC (2012). BH3 mimetics and multi-kinase inhibition in AML. Blood.

[36] Castedo M, Perfettini JL, Roumier T, Andreau K, Medema R, Kroemer G (2004). Cell death by mitotic catastrophe: a molecular definition. Oncogene.

[37] Crawford N, Chacko AD, Savage KI, McCoy F, Redmond K, Longley DB, Fennell DA (2011). Platinum resistant cancer cells conserve sensitivity to BH3 domains and obatoclax induced mitochondrial apoptosis. Apoptosis.

[38] Lamers F, Schild L, den Hartog IJ, Ebus ME, Westerhout EM, Ora I, Koster J, Versteeg R, Caron HN, Molenaar (2012). BCL2 inhibition effectively inhibits neuroblastoma tumour growth. Eur J Cancer.

[39] Rahmani M, Aust MM, Attkisson E, Williams DC, Ferreira-Gonzalez A, Grant S (2012). Inhibition of Bcl-2 antiapoptotic members by obatoclax potently enhances sorafenib-induced apoptosis in human myeloid leukemia cells through a Bim-dependent process. Blood.

[40] Konopleva M, Watt J, Contractor R, Tsao T, Harris D, Estrov Z, Bornmann W, Kantarjian H, Viallet J, Samudio I (2008). Mechanisms of antileukemic activity of the novel Bcl-2 homology domain-3 mimetic GX15-070 (obatoclax). Cancer Res.

[41] Pan J, Cheng C, Verstovsek S, Chen Q, Jin Y, Cao Q (2010). The BH3-mimetic GX15-070 induces autophagy, potentiates the cytotoxicity of carboplatin and 5-fluorouracil in esophageal carcinoma cells. Cancer Lett.

[42] Bonapace L, Bornhauser BC, Schmitz M, Cario G, Ziegler U, Niggli FK, Schäfer BW, Schrappe M, Stanulla M, Bourquin JP (2010). Induction of autophagy-dependent necroptosis is required for childhood acute lymphoblastic leukemia cells to overcome glucocorticoid resistance. J Clin Invest.

[43] Martin AP, Mitchell C, Rahmani M, Nephew KP, Grant S, Dent P (2009). Inhibition of MCL-1 enhances lapatinib toxicity and overcomes lapatinib resistance via BAK-dependent autophagy. Cancer Biol Ther.

[44] Martin AP, Park MA, Mitchell C, Walker T, Rahmani M, Thorburn A, Häussinger D, Reinehr R, Grant S, Dent P (2009). BCL-2 family inhibitors enhance histone deacetylase inhibitor and sorafenib lethality via autophagy and overcome blockade of the extrinsic pathway to facilitate killing. Mol Pharmacol.

[45] Maiuri MC, Criollo A, Tasdemir E, Vicencio JM, Tajeddine N, Hickman JA, Geneste O, Kroemer G (2007). BH3-only proteins and BH3 mimetics induce autophagy by competitively disrupting the interaction between Beclin 1 and Bcl-2/Bcl-X(L). Autophagy.

